# Hemicrania continua: towards a new classification?

**DOI:** 10.1186/1129-2377-15-8

**Published:** 2014-02-13

**Authors:** Fabio Antonaci, Ottar Sjaastad

**Affiliations:** 1 Headache Centre, C. Mondino National Institute of Neurology Foundation, IRCCS, Department of Brain and Behavioral Sciences, University of Pavia, Pavia, Italy; 2 Department of Neurology, St. Olavs Hospital, Trondheim University Hospitals, NTNU, Trondheim, Norway

**Keywords:** Hemicrania continua, Headache classification

## Abstract

Hemicrania continua (HC) was described and coined in 1984 by Sjaastad and Spierings. Later cases, carrying this appellation, should conform to the original description. The proposed classification criteria (ICHD 3rd edition beta version) for HC focus e.g. on localized, autonomic and “vascular” features. Such features do, however, not belong to the core symptomatology of HC and should accordingly be removed. The genuine, original HC will then re-appear.The headache that the new criteria refer to, has in an unfair and unjustified manner been given the designation HC. A revision of the proposed criteria seems mandatory.

## Findings

Hemicrania continua (HC) was described and coined in 1984 by Sjaastad and Spierings [1]. Naturally, later reported cases, carrying this appellation, should conform with the original description of this clinical constellation of symptoms and signs. Otherwise, errors will be introduced, and confusion will arise. The essence of this headache is: 1. Unilaterality of the head pain (Hemicrania), and without side alteration, at that. 2. Continuous head pain (continua). 3. A generally moderate, but somewhat fluctuating pain, the pain only rarely approaching a high intensity level. Thus, most patients were able to work, even when the pain was stronger than usual. The patients had generally not contemplated suicide. Nocturnal awakenings occurred, but generally only rarely and during exacerbations. 4. And then the most spectacular, single factor: the effect of indomethacin, which is obligatory and absolute- generally with small dosages, i.e. 50–75 mg per day (not more) -- In other words, patients who do not fulfill the indomethacin criteria, are not candidates for this headache category. It was already from the early phase evident that there were two temporal patterns: a primary chronic form; and another one with an initial, remitting pattern, that could last for some time, but with time generally developing into a continuous form.

The first two cases [1] were the prototypes of Hemicrania continua.—Throbbing was generally rarely present, appearing only during exacerbations. Facial/ forehead sweating and miosis are NOT parts of this picture. That these signs are included in the new criteria (ICHD 3^rd^ edition beta version) [2] seems to be a misunderstanding. The same goes for much of what is written under: “Description”. It also concerns the restlessness. This should not be used as a criterion in HC, as it is a typical feature of Cluster headache. Therefore, the proposal that the criteria “2. a sense of restlessness or agitation, or aggravation of the pain by movement” may be sufficient (in absence of other autonomic symptoms and signs) for the HC diagnosis seems to be another misunderstanding. And then, fundamental changes in the total concept of HC were brought on, after which HC easily could be confounded with similar, but nevertheless essentially different headaches. The original clinical structure of HC remains; the headache itself had not changed. That leaves only one possibility open: it was the clinicians’ concept of reality that had changed; the essence of HC had probably been tampered with. The changes that were introduced, were so fundamental that the original cases [1] no more fulfilled the original criteria. This situation is not acceptable.

What had happened? The development may seem to be like this:

It was claimed that although indomethacin seemed to play a role in HC treatment, the effect was not always absolute, and it could even be entirely lacking [3]. And much larger doses might be needed to obtain effect. The proportion of non-responders seemed to increase. In the end, a series of non-responders was published: “Hemicrania continua is not that rare” [4,5]. It is self-evident that if strict and pure criteria are conscientiously loosened, more patients will fit into this category. It was claimed that these patients nevertheless had the “phenotype” of HC. This did not stand to reason. Indomethacin response- absolute/ close to absolute- is a cornerstone in the diagnosis of HC. We have no doubt whatsoever that cases like the described ones exist. But they are not cases of HC. They have little to do with HC. They are rather cases of what we have termed: “Non-indomethacin responsive chronic hemicrania” or: NIRCH [6,7]. This group is probably larger than HC itself; it may even be much larger. This category may even contain various sub-groups. Also for that reason, this headache category should be given another appellation. It may be a promising task for young researchers to explore this field.

Actually, indomethacin mean requirement is generally rather low, i.e. around 75 mg per day. During exacerbations, the dosage may have to be enlarged to 100 or even 150 mg for a day or some days. Such periods should be kept as short-lasting as possible, since indomethacin is a potentially dangerous drug. It is, therefore, with great surprise that one reads that the recommended dosages should be “at least 150 mg daily and increased if necessary up to 225 mg daily”. The recommended, initial test-dose by “the Indotest” [8] is 50 mg intramuscularly. A dosage of 100 mg does not render any major advantage. The dosage should NOT, as recommended by the committee, be 100–200 mg. With such dosages, one does not test the specific qualities of indomethacin in this headache any more then one tests general analgesic properties, and that is not what one is searching. It would eventually give a false indication of indomethacin effect and, in the long term, such high dosages may even endanger the patient’s life. The idea behind indomethacin therapy must be to try to help the patient not to hurt him. This should be self-evident, and the same reasoning is valid for CPH, where similar dosages are recommended [2]. If high dosages are being employed during the initial trial and during the initial period of therapy, many patients will develop intolerance and side effects and eventually shy away from the further use of indomethacin.

Somewhat later, it was proposed that “migrainous/ vascular components” were integral parts of HC such as nausea/vomiting and a pulsating quality of the pain. It may also be noteworthy that in the description of this headache, unilaterality is mentioned, but not whether it is side-locked or not. This represents a further step away from the HC-picture and a major one. Together, they will wipe out the essence of the HC-picture. Vomiting may be an insignificant -and rare- part of the HC exacerbation. Vomiting does not belong to the core symptomatology of HC. It may even, partially be a by-product of drugs. It should also in this connection be pointed out that many cases published under the category HC, sail under false colors. Reviews and statistics based on such cases will not render correct figures for the various variables. The vascular ingredient story is a most unwanted and unjustified proposal that eventually might lead to a further alienation from the genuine picture of HC.

The claim that facio-cephalic autonomic symptoms can be part of the picture makes it utterly demanding to recognize the original picture of HC. Forehead/facial sweating is not part of the HC-picture. Nor is miosis. To include them among the HC criteria is actually a considerable blunder. Pupillary changes are, on the other hand, a frequent accompaniment of NIRCH [6,7].

An indomethacin-responsive hemicrania, characterized by continuous pain is most likely a case of HC, For headaches that do not fulfill the criteria of HC, one should carefully try to re-categorize them or eventually find new categories. Surprisingly, all the references to the original work [9] are erased in the new edition of the criteria [2]. As already pointed out: these are the original, genuine criteria. As for the HC type: remitting subtype (i.e. 3.4.1) [2], as regards remissions, it is not a question of “at least one day”. Remissions last for months to years. We have time and again tried to emphasize these points of view, without overwhelming response. However, “Gutta cavat lapidem, non vi, sed saepe cadendo”.

The whole situation appears to be far from ideal. There may seem to be two possible ways out of the present predicament.

I. If this headache still is to be termed Hemicrania continua in the new IHS version, the autonomic features that do not belong to the core symptomatology of HC should be removed, and so should the “vascular” features. The genuine, original HC will then re-appear.

II. An appellation other than HC should be found for the headache variant depicted in the new criteria. The headache that these criteria refers to, has in an unfair and unjustified manner been given the designation HC. The presently depicted headache is hardly a disorder in its own right, but rather a mixture of various headaches, such as cluster headache, CPH, and “vascular headache” and NIRCH. In most of the cases, proper indomethacin trials have not been carried out.

## Competing interests

The authors declare that they have no competing interests.

## Authors’ contributions

FA AND OS wrote the manuscript on the basis of the literature and on personal experince in the field Both authors read and approved the final manuscript.
